# Population genetic structure and natural selection of *Plasmodium falciparum* apical membrane antigen-1 in Myanmar isolates

**DOI:** 10.1186/s12936-018-2215-7

**Published:** 2018-02-07

**Authors:** Jung-Mi Kang, Jinyoung Lee, Mya Moe, Hojong Jun, Hương Giang Lê, Tae Im Kim, Thị Lam Thái, Woon-Mok Sohn, Moe Kyaw Myint, Khin Lin, Ho-Joon Shin, Tong-Soo Kim, Byoung-Kuk Na

**Affiliations:** 1 Department of Parasitology and Tropical Medicine, and Institute of Health Sciences, Gyeongsang National University College of Medicine, Jinju, 52727 Republic of Korea; 2 BK21Plus Team for Anti-aging Biotechnology and Industry, Department of Convergence Medical Science, Gyeongsang National University, Jinju, 52727 Republic of Korea; 3 Department of Medical Research Pyin Oo Lwin Branch, Pyin Oo Lwin, Myanmar; 4 Department of Tropical Medicine and Inha Research Institute for Medical Sciences, Inha University College of Medicine, Incheon, 22212 Republic of Korea; 5 Department of Microbiology, Ajou University College of Medicine, Suwon, 16499 Republic of Korea; 6 Present Address: Department of Infection Biology, Zoonosis Research Center, School of Medicine, Wonkwang University, Iksan, 54538 Republic of Korea; 7 Present Address: Planning and Management Division, Nakdonggang National Institute of Biological Resources, Sangju, 37242 Republic of Korea

**Keywords:** *Plasmodium falciparum*, Apical membrane antigen-1, Genetic diversity, Natural selection, Myanmar

## Abstract

**Background:**

*Plasmodium falciparum* apical membrane antigen-1 (PfAMA-1) is one of leading blood stage malaria vaccine candidates. However, genetic variation and antigenic diversity identified in global PfAMA-1 are major hurdles in the development of an effective vaccine based on this antigen. In this study, genetic structure and the effect of natural selection of PfAMA-1 among Myanmar *P. falciparum* isolates were analysed.

**Methods:**

Blood samples were collected from 58 Myanmar patients with falciparum malaria. Full-length PfAMA-1 gene was amplified by polymerase chain reaction and cloned into a TA cloning vector. PfAMA-1 sequence of each isolate was sequenced. Polymorphic characteristics and effect of natural selection were analysed with using DNASTAR, MEGA4, and DnaSP programs. Polymorphic nature and natural selection in 459 global PfAMA-1 were also analysed.

**Results:**

Thirty-seven different haplotypes of PfAMA-1 were identified in 58 Myanmar *P. falciparum* isolates. Most amino acid changes identified in Myanmar PfAMA-1 were found in domains I and III. Overall patterns of amino acid changes in Myanmar PfAMA-1 were similar to those in global PfAMA-1. However, frequencies of amino acid changes differed by country. Novel amino acid changes in Myanmar PfAMA-1 were also identified. Evidences for natural selection and recombination event were observed in global PfAMA-1. Among 51 commonly identified amino acid changes in global PfAMA-1 sequences, 43 were found in predicted RBC-binding sites, B-cell epitopes, or IUR regions.

**Conclusions:**

Myanmar PfAMA-1 showed similar patterns of nucleotide diversity and amino acid polymorphisms compared to those of global PfAMA-1. Balancing natural selection and intragenic recombination across PfAMA-1 are likely to play major roles in generating genetic diversity in global PfAMA-1. Most common amino acid changes in global PfAMA-1 were located in predicted B-cell epitopes where high levels of nucleotide diversity and balancing natural selection were found. These results highlight the strong selective pressure of host immunity on the PfAMA-1 gene. These results have significant implications in understanding the nature of Myanmar PfAMA-1 along with global PfAMA-1. They also provide useful information for the development of effective malaria vaccine based on this antigen.

## Background

Global incidence and mortality of malaria have decreased in recent years, but malaria is still a great public health concern. In 2015, there were approximately 212 million clinical cases of malaria with an estimated 429,000 deaths [1]. The challenge for malaria control and elimination has increased due to the spread of parasites with resistance to anti-malarial drugs and insecticide-resistant *Anopheles* mosquitoes. The lack of effective vaccines is one of the major obstacles in combating malaria. Therefore, development of efficacious vaccine is urgently needed for malaria control. Up to date, several candidate proteins including circumsporozoite protein (CSP), Duffy-binding protein (DBP), merozoite surface protein-1 (MSP-1), apical membrane antigen-1 (AMA-1), and thrombospondin related anonymous protein (TRAP) have been tested for their potentials as candidate antigens to develop effective vaccines [2]. The majority of these antigens are expressed either in pre-erythrocytic and erythrocytic stages [2, 3]. However, genetic polymorphisms identified in these parasite antigens are great hurdles to develop effective vaccines since they generate a variant-specific immune response which is less effective against parasites with other genetic variants [4–6].

Apical membrane antigen-1 (AMA-1) is a 83-kDa type I integral membrane protein that is mainly expressed in the merozoite and sporozoite [7, 8]. Biological function of AMA-1 is not clearly understood yet, but its stage specific expression and localization suggest that the protein might play a crucial role in invasion of erythrocytes and hepatocytes by malaria parasites [8–11]. Structure analysis of AMA-1 revealed that this protein consists of a signal sequence, a cysteine-rich ectodomain, a conserved cytoplasmic region and a transmembrane region [12]. The ectodomain of AMA-1 is further subdivided into three domains, domains I, II, and III. The ectodomain AMA-1 is very immunogenic and natural immune responses against the domain have been reported in patients exposed to *Plasmodium falciparum* and *Plasmodium vivax* [13–16]. Immunization with AMA-1 can produce antibodies to effectively inhibit the invasion of erythrocyte by malaria parasite and confer protective immune responses [15, 17]. As a result, AMA-1 is a leading blood stage vaccine candidate for malaria control [16, 18, 19]. Several studies have shown that anti-AMA-1 antibodies confer protective immunity in adults living in malaria-endemic areas [20, 21]. However, these antibodies recognize either conserved or allele-specific epitopes of AMA-1, resulting limited protection against different alleles [22]. AMA-1 is known to be less variable than other malaria vaccine candidate antigens such as MSPs or CSP, but AMA-1 also possesses genetic diversity among global malaria parasites. A high rate of polymorphisms has been identified in domain I of AMA-1 and this region appears to be a major target of anti-AMA-1 protective antibodies [23–28]. To design an efficient and protective malaria vaccine, it is essential to monitor genetic variations of vaccine candidate antigens among global malaria isolates circulating in endemic areas [29].

The incidence of malaria in Myanmar has decreased in recent years. However, Myanmar still accounts for more than half of the malaria cases and approximately three quarters of the malaria deaths in the Greater Mekong Subregion [30]. Although *P. falciparum* cases have decreased in recent years [1, 31], this species is still the most critical concern for malaria control and prevention in Myanmar with the emergence of artemisinin resistance [32]. In this study, population genetic structure and natural selection of *P. falciparum* AMA-1 (PfAMA-1) in Myanmar *P. falciparum* isolates were analysed.

## Methods

### Blood samples and ethics

**Fig. 1:**
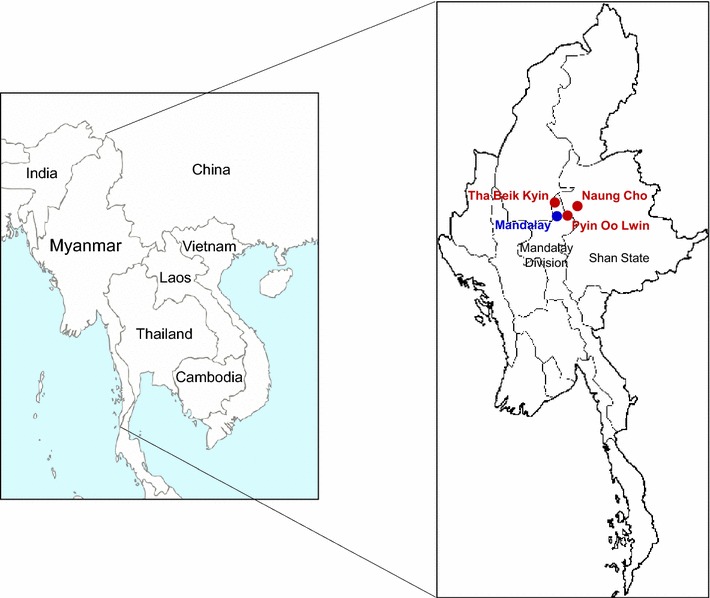
Map of the study sites. Community-based survey was performed in seven villages in Naung Cho, Shan State, and Pyin Oo Lwin and Tha Beik Kyin, Mandalay Division, Upper Myanmar between July to December 2015. Blood samples were collected from *P. falciparum* infected patients and used in this study

Blood samples used in this study were obtained from *P. falciparum* infected patients who resided in rural villages located in Naung Cho, Shan State, and Pyin Oo Lwin and Tha Beik Kyin, Mandalay Division, Upper Myanmar between July to December 2015 (Fig. 1). Malaria transmission was heterogeneous and seasonal in these areas and most malaria cases occurred with the peak during and just after the rainy season. The major ethnic groups in these regions were Bamar and Shan. Community-based survey was performed in 825 residents in 7 villages of the areas and *P. falciparum* infection was confirmed by Giemsa-stained thick and thin blood smear examination. The average age of patients was 34.7 years old ranged from 13 to 60 years. Two or three ml of venous blood was collected from the *P. falciparum* infected patients in EDTA tubes before drug treatment for further molecular analysis. All *P. falciparum* positive samples were further confirmed by polymerase chain reaction (PCR) targeting 18S ribosomal RNA (rRNA) gene [31, 33]. The use of blood samples in this study was approved by the Ministry of Health, Myanmar (Approval Number: 97/Ethics 2015). It was also approved by Biomedical Research Ethics Review Board of Inha University College of Medicine, Republic of Korea (Approval Number: INHA 15-013). Written consent was obtained from each individual prior to blood collection.

### Amplification and sequencing analysis of PfAMA-1

Genomic DNA of parasite was extracted from 200 µl of whole blood sample of patients using QIAamp DNA Blood Kit (Qiagen, Hilden, Germany) following the manufacturer’s instruction. Full-length PfAMA-1 gene was amplified by PCR. PfAMA-1 gene-specific primers were designed based on complete coding sequences of PfAMA-1 of *P. falciparum* isolates 3D7 (GenBank Accession No.: U65407) and FC27 (GenBank Accession No.: M27133). Forward and reverse primers were 5′-GTACTTGTTATAAATTGTACA-3′ and 5′-TTTTAGCATAAAAGAGAAGC-3′, respectively. Thermal cycling parameters for PCR were as follows: one cycle of an initial denaturation at 94 °C for 5 min, 30 cycles of 94 °C for 1 min, annealing at 55 °C for 1 min and extension at 72 °C for 2 min, followed by a final extension step at 72 °C for 10 min. Nested PCR with primers 5′-ACAAAAATGAGAAAATTATACTGC-3′ and 5′-TTAATAGTATGGTTTTTCCATCAGAAC-3′ was performed with the same amplification condition, if necessary. To minimize nucleotide mis-incorporation into sequences during PCR amplification, Ex Taq DNA polymerase (Takara, Otsu, Japan) with proof-reading activity was used in all PCR. Each PCR product was analysed by electrophoresis on 1% agarose gels. The resulting PCR product was cut from the gel followed by gel purification. It was then cloned into T&A cloning vector (Real Biotech Corporation, Banqiao City, Taiwan). Each ligation mixture was transformed into *Escherichia coli* DH5α competent cells. Colony PCR was performed with the nested PCR primers to identify positive clones with appropriate insert. Nucleotide sequences of cloned PCR product were subjected to automatic DNA sequencing using M13 forward and M13 reverse primers. Sequencing was also carried out using two additional specific internal primers (5′-CAGGGAAATGTCCAGTATTTGGTA-3′ and 5′-TTCCATCGACCCATAATCCG-3′) to obtain clear sequences for central part of PfAMA-1. At least two clones from each isolate were sequenced to ensure sequencing accuracy. Some isolates underwent three or fourfold sequence coverage to confirm the existence of rare polymorphisms. These nucleotide sequences are deposited at GenBank under Accession Numbers KU893276–KU893333.

### Nucleotide sequence polymorphism analysis and neutrality test

Nucleotide and deduced amino acid sequences of PfAMA-1 were analysed using EditSeq and SeqMan programs in DNASTAR package (DNASTAR, Madison, WI, USA). Nucleotide sequence polymorphism analysis was conducted for 58 PfAMA-1 sequences. Numbers of segregating sites (S), haplotypes (H), haplotype diversity (Hd), nucleotide diversity (π), and average number of pair-wise nucleotide differences within a population (*K*) were estimated using DnaSP ver. 5.10.00 [34]. Value of π was calculated to estimate step-wise diversity throughout the full-length PfAMA-1 based on a sliding window of 100 bases with a step size of 25 bp. Values of non-synonymous (dN) and synonymous (dS) substitutions were estimated and were compared using Z test (*P* < 0.05 was considered significant) in MEGA4 program [35] based on method of Nei and Gojobori [36] with Jukes and Cantor correction. The Tajima’s D value [37] and Fu and Li’s D and F values [38] were analysed using DnaSP ver. 5.10.00 to evaluate neutral theory of evolution [34]. Recombination parameter (R), which included the effective population size and probability of recombination between adjacent nucleotides per generation, and minimum number of recombination events (Rm) were analysed by DnaSP ver. 5.10.00 [34]. Linkage disequilibrium (LD) between different polymorphic sites was computed based on the R^2^ index using DnaSP ver. 5.10.00 [34].

### Population diversity of PfAMA-1 among global *P. falciparum* isolates

Genetic diversity of PfAMA-1 in global *P. falciparum* isolates was analysed. Parasite populations from Ghana (n = 37, AB715698–AB715734), Papua New Guinea (PNG: n = 90, AB715870–AB715959), Philippines (n = 55, AB715815–AB715869), Solomon Islands (n = 50, AB715960–AB716009), Tanzania (n = 62, AB715636–AB715679), Thailand (n = 80, AB715735–AB715814), and Vanuatu (n = 85, AB716010–AB716094) were included in this analysis. These publically available sequences covered full-length sequences of PfAMA-1. Nucleotide sequence polymorphism analysis and neutrality test for each population were performed using DnaSP ver. 5.10.00 [34] and MEGA4 program [35] as described above. Genetic differentiation among parasite populations was calculated based on fixation index (Fst) to estimate pairwise DNA sequence diversity between and within populations using DnaSP ver. 5.10.01 [34]. To investigate relationships among PfAMA-1 haplotypes, the haplotype network for a total of 517 PfAMA-1 sequences including 58 Myanmar sequences and the 459 publically available sequences from Ghana, PNG, Philippines, Solomon Islands, Tanzania, Thailand, and Vanuatu was constructed using the program NETWORK version 5.0.0.3 with the Median Joining algorithm [39]. To assess whether genetic diversity in PfAMA-1 within *P. falciparum* isolates were associated with host’s immune pressure, genetic diversity in predicted B-cell epitopes, intrinsically unstructured/disordered regions (IUR), and RBC binding regions in global PfAMA-1 were analysed [40, 41]. Nucleotide diversity and natural selection of each region were also analysed using DnaSP ver. 5.10.00 [34], as described above.

## Results

### Sequence polymorphism in Myanmar PfAMA-1 sequences

**Fig. 2:**
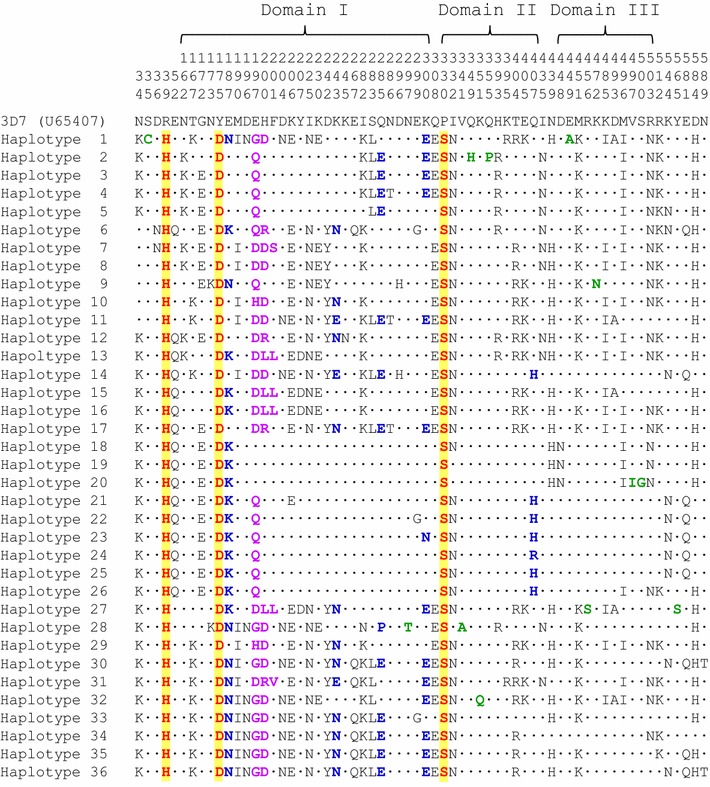
Amino acid sequence polymorphism of the PfAMA-1 in Myanmar *Plasmodium falciparum* isolates. Thirty-seven haplotypes of Myanmar PfAMA-1 classified by nucleotide sequences were further classified into 36 distinct haplotypes based on amino acid sequences. Amino acid sequences of 36 haplotypes of Myanmar PfAMA-1 were compared to PfAMA-1 of 3D7 (GenBank Accession No.: U65407). Identical amino acid residues are indicated by dots. A total of 78 non-synonymous SNPs identified in 58 Myanmar PfAMA-1 resulted in amino acid changes at 64 positions. These amino acid changes were not evenly distributed in each PfAMA-1 haplotype. However, sequences clustered into an individual haplotype shared common amino acid changes. Conserved amino acid changes identified in all Myanmar PfAMA-1 sequences are presented as bold red with yellow background. Tri-morphic amino acid changes are presented as bold blue. Tetra- or penta-morphic amino acid changes are marked as bold purple. Rare amino acid changes identified are marked in bold green

Fifty-eight PfAMA-1 sequences with 37 different haplotypes were obtained from Myanmar *P. falciparum* isolates. Nucleotide sequence analysis of these 58 PfAMA-1 sequences compared to PfAMA-1from 3D7 (GenBank Accession Number: U65407) revealed 83 single nucleotide polymorphisms (SNPs) in Myanmar PfAMA-1 sequences, including 5 synonymous SNPs and 78 non-synonymous SNPs. These non-synonymous SNPs resulted in amino acid changes at 64 positions in 58 Myanmar PfAMA-1 sequences, including 56 di-morphic amino acid changes and 8 tri-, tetra-, or penta-morphic amino acid changes (Fig. 2). Most amino acid changes were identified in domain I (28 amino acid changes), domain II (11 amino acid changes), and domain III (11 amino acid changes). Although these amino acid changes were distributed throughout each Myanmar PfAMA-1 haplotype, three amino acid changes (R39H, Y175D, and P330S) were fixed in all Myanmar PfAMA-1 sequences (Fig. 2). Of the eight tri-, tetra-, or penta-morphic amino acid changes, seven (E187K/N, E197Q/G/H/D, H200L/D/R, F201L/S/V, M243N/E, Q285E/P, and K300E/N) were found in domain I while the other one (Q407H/R) was found in domain II. Of the 56 di-morphic amino acid changes identified in Myanmar PfAMA-1, 44 have been previously reported in PfAMA-1 from other geographical *P. falciparum* isolates. However, the remaining 12 changes (S35C, N297T, V341A, Q349H, K351Q, Q355P, E449A, R456S, K472N, V497I, S500G, and Y565S) are novel that have not been reported previously.

### Amino acid polymorphisms in Myanmar PfAMA-1 compared to global PfAMA-1

**Fig. 3:**
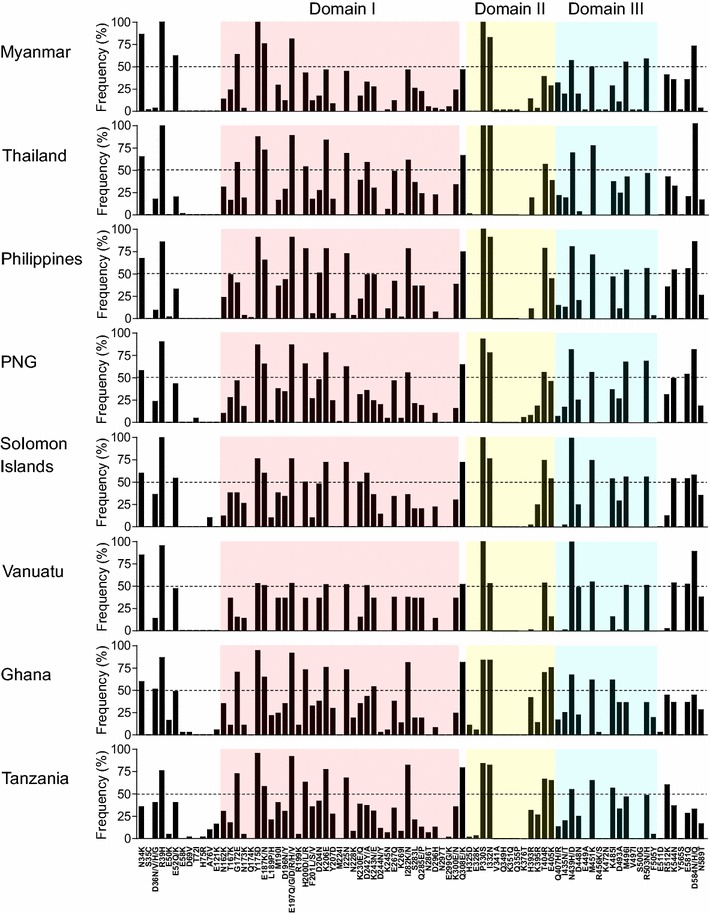
Comparison of amino acid polymorphisms of PfAMA-1 among global *Plasmodium falciparum* isolates. Positions and frequencies of amino acid changes found in PfAMA-1 of global *P. falciparum* isolates are compared. Each domain is represented by different color; Domain I (pink), domain II (yellow), and domain III (sky blue)

When patterns of amino acid polymorphisms identified in Myanmar PfAMA-1 were compared to those of PfAMA-1 from other countries, similar but not identical polymorphic patterns were observed. Most amino acid changes identified in global PfAMA-1 were found in domains I and III. Amino acid changes were also found in domain II, 5ʹ-terminal (5ʹ-T), and 3ʹ-terminal (3ʹ-T) regions (Fig. 3). Overall patterns of amino acid changes observed in global PfAMA-1 were similar to each other. However, frequencies of each amino acid change differed by country. The most notable amino acid changes identified in Myanmar PfAMA-1 were that three amino acid changes (R39H, Y175D and P330S) were fixed in all Myanmar PfAMA-1 sequences, even though their frequencies were also high in PfAMA-1 of other global isolates. Frequencies of R39H variation in other global isolates were as follow: Thailand, 100%; Philippines, 85.5%; PNG, 90.0%; Solomon Islands, 100%; Vanuatu, 95.3%; Ghana, 86.5%; Tanzania, 75.8%. The frequency of Y175D variation was also identified high in PfAMA-1 sequences from Thailand (87.5%), Philippines (90.9%), PNG (86.7%), Solomon Islands (76.0%), Ghana, (94.6%), and Tanzania, (95.2%) except for Vanuatu (52.9%). Substitution rate of P330S was also high in PfAMA-1 from all countries: Thailand, 100%; Philippines, 85.5%; PNG, 93.3%; Solomon Islands, 100%; Vanuatu, 100%; Ghana 83.8%; Tanzania 83.9%. High frequencies of N34K (86.2%), E52Q/K (62.1%), G172E (63.8%), E187K/N (75.8%), E197Q/G/D/H (81.1%), I332N (82.8%), and D584H (70.7%) variations were also observed in Myanmar PfAMA-1. These seven amino acid changes also showed high level of polymorphic patterns in global PfAMA-1 sequences. However, their frequencies varied by country. Meanwhile, S35C, N297T, V341A, Q349H, K351Q, Q355P, E449A, R456S, K472N, V497I, S500G, and Y565S were uniquely identified in Myanmar PfAMA-1, although their substitution frequencies were very low. Six amino acid changes (E50K, D69V, E121K, R199K, N228K, and E328K) were common in African PfAMA-1 sequences (Ghana and Tanzania). They were not identified or very rarely identified in PfAMA-1 sequences from other countries.

### Nucleotide diversity and natural selection of Myanmar PfAMA-1

Nucleotide diversity and natural selection of PfAMA-1 of these 58 Myanmar *P. falciparum* isolates were analysed. The *K* value for full-length PfAMA-1 of Myanmar samples was 19.886 (Table 1). The highest nucleotide differences was found at domain I (*K* = 9.796) while the lowest was found at 5ʹ-T (*K* = 1.253). Haplotype diversity for full-length PfAMA-1 sequences of these 58 isolates was 0.948 ± 0.019. This value was higher at domain I (0.912 ± 0.024) than that at domains III (0.871 ± 0.024) or domain II (0.860 ± 0.023). The π value of the full-length PfAMA-1 for Myanmar isolates was 0.01064 ± 0.00051. Analysis of the π values for the 5ʹ-T, domain I, domain II, domain III, and 3ʹ-T revealed that most nucleotide diversity was concentrated at domains I and III. The π value for each fragment was: 0.00282 ± 0.00027 for 5ʹ-T, 0.02120 ± 0.00161 for domain I, 0.00752 ± 0.00057 for domain II, 0.01629 ± 0.00080 for domain III, and 0.00581 ± 0.00047 for 3ʹ-T (Table 1). To examine whether natural selection might have contributed to generation of PfAMA-1 diversity in Myanmar *P. falciparum* population, the value of dN − dS was estimated using the Nei and Gojobori’s method [36]. The value of dN − dS for full-length PfAMA-1 was 0.01076, suggesting that balancing natural selection might have occurred in the PfAMA-1 of Myanmar *P. falciparum* population. Considering high positive dN − dS values for domains I (0.02516) and III (0.01484), these domains might be main regions under balancing natural selection forces. The estimated Tajima’s D was 0.57189 (*P* > 0.10) (Table 1). When Tajima’s D value was analysed for each domain, domain I and domain III showed higher positive Tajima’s D values compared to other domains.

**Table 1:** Estimates of DNA sequence polymorphism and tests of neutrality at PfAMA-1 among *Plasmodium falciparum* Myanmar isolates

Fragment	Segregating sites (S)	Singleton variable sites	Parsimony informative sites	Total no. of mutations	*K*	H	Hd ± SD	π ± SD	dN − dS	Tajima’s D
5ʹ-Terminal	5	1	4	5	1.253	8	0.702 ± 0.052	0.00282 ± 0.00027	− 0.00227	0.37596 (*P* > 0.1)
Domain I	37	4	33	38	9.796	27	0.912 ± 0.024	0.02120 ± 0.00161	0.02516	0.64221 (*P* > 0.1)
Domain II	12	4	8	12	2.234	12	0.860 ± 0.023	0.00752 ± 0.00057	0.00794	− 0.39855 (*P* > 0.1)
Domain III	13	5	8	13	3.275	14	0.871 ± 0.024	0.01629 ± 0.00080	0.01484	0.48627 (*P* > 0.1)
3ʹ-Terminal	8	2	6	8	1.987	10	0.761 ± 0.027	0.00581 ± 0.00047	0.00658	0.39702 (*P* > 0.1)
Full	78	16	62	79	19.886	37	0.948 ± 0.019	0.01064 ± 0.00051	0.01076	0.57189 (*P* > 0.1)

*K*, average number of pairwise nucleotide differences; H, number of haplotypes; Hd, haplotype diversity; π, observed average pairwise nucleotide diversity; dN, rate of non-synonymous mutations; dS, rate of synonymous mutations

### Nucleotide diversity and natural selection of PfAMA-1 in global isolates

**Fig. 4:**
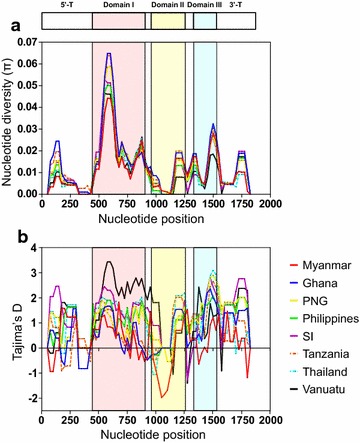
Nucleotide diversity and natural selection of global PfAMA-1. **a** Nucleotide diversity. Sliding window plot analysis showed nucleotide diversity (π) values across global PfAMA-1 sequences analysed. A window size of 100 bp and a step size of 25 bp were used. **b** Natural selection. Sliding window calculation of Tajima’s D statistic was performed for global PfAMA-1. A window size of 100 and a step size of 25 were used. Myanmar, red line; Ghana, blue line; Papua New Guinea (PNG), yellow line; Philippines, green line; Solomon Islands (SI), purple; Tanzania, dotted red line; Thailand, dotted blue line; and Vanuatu, black line

Nucleotide diversity of PfAMA-1 among global isolates, including Ghana, PNG, Philippines, Solomon Islands, Tanzania, Thailand, and Vanuatu, were analysed and compared to Myanmar PfAMA-1. *K* values in African PfAMA-1 (Ghana, *K* = 26.261; Tanzania, *K* = 25.444) were higher than those of Asian and Pacific PfAMA-1. Nucleotide diversity across the full-length PfAMA-1 from different geographical areas also slightly differed by geographical areas (Table 2). The level of nucleotide diversity across PfAMA-1 in Myanmar *P. falciparum* isolates (π = 0.01064) was lower than that for PfAMA-1 from Ghana (π = 0.01405), PNG (π = 0.01226), Tanzania (π = 0.01361), or Solomon Islands (π = 0.01179), but similar to that for PfAMA-1 from Thailand (π = 0.01103), Philippines (π = 0.01136), or Vanuatu (π = 0.01012). A sliding window plot of π revealed that PfAMA-1 from different geographical areas shared highly similar patterns of nucleotide diversity through their sequences with two peaks at domains I and III. The maximum diversity was found at domain I (Fig. 4a). All PfAMA-1 sequences from different countries showed positive Tajima’s D values, suggesting that balancing selection of global PfAMA-1 (Table 2). A sliding window plot analysis also showed that global PfAMA-1 had similar pattern for Tajima’s D across the gene, albeit some differences were identified among PfAMA-1 with different geographical origins (Fig. 4b).

**Table 2:** Estimates of DNA sequence polymorphism and tests of neutrality at PfAMA-1 among global *Plasmodium falciparum*

Isolates	Segregating sites (S)	Singleton variable sites	Parsimony informative sites	Total no. of mutations	*K*	H	Hd ± SD	π ± SD	dN/dS	Tajima’s D	Fu and Li’s D	Fu and Li’s F
Myanmar (n = 58)	78	16	62	79	19.886	37	0.948 ± 0.019	0.01064 ± 0.00053	6.524	0.57189 (*P* > 0.1)	0.16773 (*P* > 0.1)	0.38506 (*P* > 0.1)
Thailand (n = 80)	58	5	53	61	20.621	21	0.920 ± 0.013	0.01103 ± 0.00020	6.698	2.22215 (*P* < 0.05)	0.95723 (*P* > 0.1)	1.75160 (*P* < 0.05)
Philippines (n = 55)	61	3	58	62	21.232	19	0.916 ± 0.019	0.01136 ± 0.00027	7.200	1.95452 (0.1 > *P* > 0.05)	1.61226 (*P* < 0.05)	2.07959 (*P* < 0.02)
PNG (n = 90)	68	2	66	75	22.923	28	0.954 ± 0.007	0.01226 ± 0.00016	7.497	1.80824 (0.1 > *P* > 0.05)	1.67585 (*P* < 0.05)	2.07602 (*P* < 0.02)
Solomon Islands (n = 50)	56	3	53	57	22.044	9	0.862 ± 0.021	0.01179 ± 0.00027	7.023	2.54552 (*P* < 0.05)	1.54470 (*P* < 0.05)	2.28042 (*P* < 0.02)
Vanuatu (n = 85)	50	6	44	50	18.905	5	0.633 ± 0.028	0.01012 ± 0.00031	6.471	2.89729 (*P* < 0.01)	0.86419 (*P* > 0.1)	2.00393 (*P* < 0.05)
Ghana (n = 37)	75	7	68	85	26.261	32	0.992 ± 0.008	0.01405 ± 0.00034	7.423	1.06738 (*P* > 0.1)	1.21791 (*P* > 0.1)	1.38496 (*P* > 0.1)
Tanzania (n = 62)	81	8	73	89	25.444	47	0.989 ± 0.005	0.01361 ± 0.00021	8.135	1.18188 (*P* > 0.1)	1.16354 (*P* > 0.1)	1.40203 (*P* > 0.1)

*K*, average number of pairwise nucleotide differences; H, number of haplotypes; Hd, haplotype diversity; π, observed average pairwise nucleotide diversity; dN, rate of non-synonymous mutations; dS, rate of synonymous mutations

### Recombination and linkage disequilibrium

**Fig. 5:**
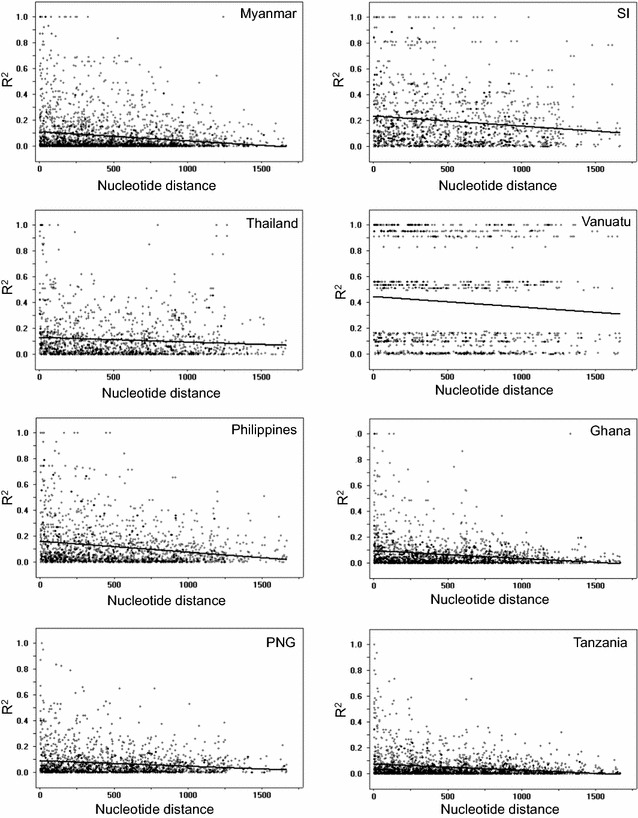
Recombination event in global PfAMA-1. Linkage disequilibrium (LD) plot showed non-random associations between nucleotide variants in PfAMA-1 at different polymorphic sites. R^2^ values were plotted against nucleotide distance using a two-tailed Fisher’s exact test for statistical significance. *PNG* Papua New Guinea, *SI* Solomon Islands

The minimum number of recombination events between adjacent polymorphic sites (Rm) for Myanmar PfAMA-1 was estimated to be 24. The R values between adjacent sites (Ra) and per gene (Rb) were 0.0141 and 26.3, respectively. Possible recombination events were also identified in global PfAMA-1. The highest R values were predicted for African PfAMA-1 (Ghana and Tanzania), while the lowest R values were predicted for Vanuatu PfAMA-1 (Table 3). LD index (R^2^) for global PfAMA-1 also decreased with increasing distance across the gene (Fig. 5).

**Table 3:** Comparison of recombination events between global PfAMA-1

	Ra	Rb	Rm
Myanmar	0.0141	26.3	24
Ghana	0.0921	172	27
PNG	0.0500	83.4	28
Philippines	0.0218	40.8	17
Solomon Islands	0.0120	22.5	14
Tanzania	0.0915	171	28
Thailand	0.0243	45.3	20
Vanuatu	0.0010	1.8	9

The R and Rm were estimated excluding the sites containing alignment gaps or those segregating for three nucleotides. The R was computed using R = 4Nr, where N is the population size and r is the recombination rate per sequence (per gene) n, number of isolates; Ra, recombination parameter between adjacent sites; Rb, recombination parameter for entire gene; Rm, minimum number of recombination events between adjacent sites

### Haplotype network analysis

**Fig. 6:**
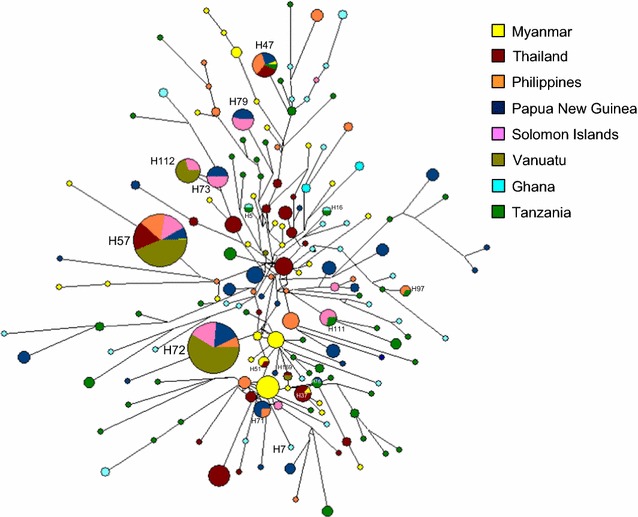
Network analysis of global PfAMA-1 haplotypes. Haplotype network was constructed using the program NETWORK version 5.0.0.3 with the Median Joining algorithm. A total of 517 PfAMA-1 sequences were analysed. The size of each node reflected the frequency of a particular haplotype. The lengths of the lines connecting the nodes, measured from their centers, were in proportion to the number of base pair substitutions separating the haplotypes. Color of each node indicated different country

Haplotype network analysis of PfAMA-1 haplotypes from global *P. falciparum* populations showed a dense network with extremely complex relationships (Fig. 6). A total of 174 distinct haplotypes were identified in 517 PfAMA-1 sequences analysed, of which 33.9% was singleton. Haplotype prevalence ranged from 0.19 to 13.7%. The most prevalent haplotype was haplotype 57 (H57) with a frequency of 13.7%. This haplotype was the only one shared by populations from six different Asian and Pacific countries (Myanmar, Thailand, Philippines, PNG, Solomon Islands, and Vanuatu). Haplotype 72 (H72) was another major haplotype with a high prevalence (13.1%) and was composed with Pacific populations. Four haplotypes, H71, H73, H79, and H112, were also admixed ones by Pacific populations. Meanwhile, two haplotypes, H5 and H16, were composed solely of African populations, Ghana and Tanzania. Only four haplotypes, H47, H76, H97, and H111, were haplotypes of African populations mixed with Asian or Pacific populations. Haplotype with sequence identical to 3D7 (GenBank Accession No.: U65407) and FVO (GenBank Accession No.: AJ277646) was H7 and H57, respectively.

### Nucleotide differentiation among global PfAMA-1

To investigate the degree of gene flow and genetic differentiation among global PfAMA-1 sequences, Fst values were evaluated for full-length PfAMA-1 sequences from different geographical *P. falciparum* populations deposited at GenBank (Table 4). Fst values between different geographical PfAMA-1 populations varied from 0.00019 (between Ghana and Tanzania) to 0.14141 (between Vanuatu and Tanzania).

**Table 4:** Pairwise F_ST_ estimates for PfAMA-1

	Myanmar	Thailand	Philippines	PNG	Solomon Islands	Vanuatu	Ghana	Tanzania
Myanmar		20.15798	20.35295	21.58878	20.68805	19.15425	22.14497	20.15798
Thailand	0.06073		20.87018	21.83959	21.16846	19.73726	22.40481	22.72679
Philippines	0.08390	0.03818		22.28146	21.61888	19.81950	23.25483	23.46398
PNG	0.05281	0.03818	0.02319		22.60884	20.97135	23.89528	23.95093
Solomon Islands	0.09605	0.06636	0.03529	0.03529		20.06783	23.83759	23.92600
Vanuatu	0.09740	0.10535	0.06689	0.06689	0.04933		21.13622	21.66300
Ghana	0.07802	0.04140	0.04167	0.03640	0.04953	0.12425		25.74924
Tanzania	0.08139	0.05865	0.05787	0.05787	0.05872	0.14141	0.00019	

Fst values are shown in the lower left quadrant and average number of pair-wise nucleotide differences between populations (K) are shown in the upper right quadrant. Fst, a measure of genetic differentiation between populations (range from 0 to + 1)

### Association between natural selection and host immune pressure

**Fig. 7:**
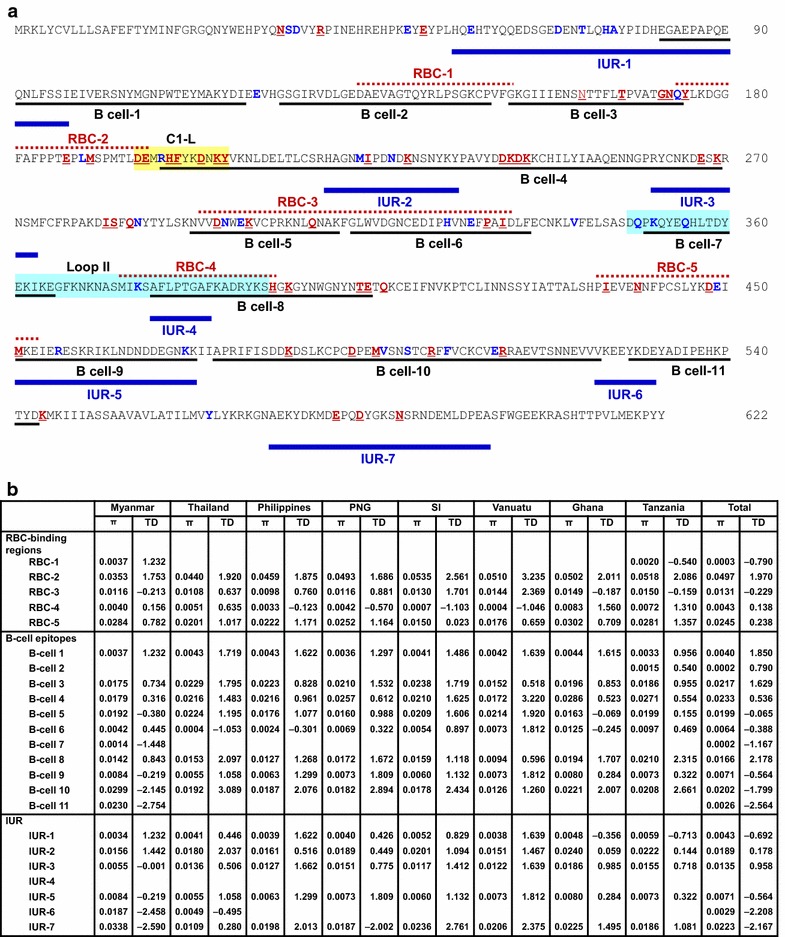
Association between natural selection and host immune pressure. **a** Positions of amino acid changes found in global PfAMA-1 and predicted RBC-binding regions, B-cell epitopes, and IUR regions. Predicted RBC-binding regions [41], B-cell epitopes, and IUR regions [40] are presented by black underlines, dotted red lines, and bold blue lines, respectively. Polymorphic amino acid residues commonly identified in global PfAMA-1 are marked as bold red with underline. The less commonly identified amino acid changes are shown as bold blue. C1-L (cluster 1 of loop I, aa: 196–207) in DI and loop II (aa: 348–392) in DII are shown in yellow and sky blue squares, respectively. **b** Nucleotide diversity and natural selection analysis. Nucleotide diversity (π) and Tajima’s D (TD) values for each RBC-binding region, B-cell epitope region, and IUR region in global PfAMA-1 were analysed using DnaSP program. *PNG* Papua New Guinea, *SI* Solomon Islands

The selective pressure of host immunity on PfAMA-1 was evaluated by analysing genetic polymorphisms in predicted RBC-binding sites, B-cell epitopes and IUR regions. Results showed that most amino acid changes caused by SNPs were found in predicted RBC-binding sites, B-cell epitopes, or IUR regions of PfAMA-1 (Fig. 7a). Of 83 amino acid changes, 66 were found in global PfAMA-1 compared to PfAMA-1 of 3D7 (GenBank Accession No.: U65407) at predicted RBC-binding sites, B-cell epitopes, or IUR regions. Among 51 commonly identified amino acid changes in global PfAMA-1, 43 were found in predicted RBC-binding sites, B-cell epitopes, or IUR regions. The 23 less commonly changed amino acids in global PfAMA-1 sequences were also found in predicted RBC-binding sites, B-cell epitopes, or IUR regions. Eight of 11 predicted B-cell epitopes were polymorphic. In particular, high levels of *π* were predicted for B-cell epitopes 3, 4, 5, 8, and 10. These regions contained major polymorphic amino acid residues in global PfAMA-1 (Fig. 7b). Tajima’s D values for the predicted B-cell epitopes 3, 4, and 8 were all positive, indicating a decrease in population size and/or balancing selection. Meanwhile, Tajima’s D values for the predicted B-cell epitopes 5 and 10 were negative (Fig. 7b). Common amino acid changes found in global PfAMA-1 were located at C1-L covering region, which is located near the hydrophobic pocket of PfAMA-1. Meanwhile, only a few less frequent amino acid changes were identified in loop II of PfAMA-1.

## Discussion

Population genetic structure and natural selection of PfAMA-1 among Myanmar *P. falciparum* isolates were analysed in this study. Most of amino acid changes identified in Myanmar PfAMA-1 were clustered at domains I and III, consistent with previous reports on PfAMA-1 from other endemic areas [40, 42–45]. Overall distribution patterns and frequencies of amino acid changes found in Myanmar PfAMA-1 were similar to those of global PfAMA-1 analysed in this study, but several differences among and between global PfAMA-1 were also identified. The implication of these substantial geographic differentiations is currently unclear. Considering that only a limited number of PfAMA-1 sequences in each geographical area were analysed in this study, these amino acid changes and their different frequencies may not be statistically significant. Indeed, many amino acid changes found in global PfAMA-1 were commonly detected and evenly distributed among global PfAMA-1 sequences, although their frequencies differed among and between populations. Further examination of PfAMA-1 nucleotide and amino acid variations in diverse PfAMA-1 populations with a larger number of global PfAMA-1 sequences would be necessary to better understand the polymorphic nature of PfAMA-1. It is also worthy to mention that Myanmar PfAMA-1 sequences analysed in this study were from *P. falciparum* isolates that collected in restricted areas of Myanmar. Therefore, nation-wide analysis of PfAMA-1 in *P. falciparum* isolates collected from various regions of Myanmar is also needed to clearly understand the genetic diversity and population structure of Myanmar PfAMA-1.

The π value for global PfAMA-1 varied, ranging from 0.01012 (Vanuatu) to 0.01405 (Ghana). The π values in African PfAMA-1 sequences (Ghana and Tanzania) were relatively higher than those in Asian or Pacific PfAMA-1 sequences. Nucleotide diversity was not evenly distributed through Myanmar PfAMA-1. The 5ʹ-T, domain II and 3ʹ-T showed low levels of nucleotide diversity, indicating that these regions might be more conserved with lower frequency of polymorphisms. Meanwhile, much higher value of nucleotide diversity was observed at domain I and domain III. Highly similar patterns of nucleotide diversity were also detected in global PfAMA-1 analysed in this study, strongly indicating that global PfAMA-1 might share highly similar nucleotide diversity across this gene. The dN − dS value for Myanmar full-length PfAMA-1 was positive, implying balancing selection in this gene. However, the dN − dS value for 5ʹ-T was negative, suggesting negative or purifying natural selection in this domain. Tajima’s D value (the statistic value predicting departure from neutrality) for Myanmar PfAMA-1 also suggested that this gene might have evolved under balancing selection. In sliding plot analysis of Tajima’s D, general patterns of Tajima’s D values across Myanmar PfAMA-1 were similar to those of global PfAMA-1, although differences among and between PfAMA-1 from different countries were also found. Regardless of theses slight differences, domains I and III and the junction between domains II and III shared very similar patterns of Tajima’s D values in global PfAMA-1, indicating that these regions might be dominant targets for host immune response. Fu and Li’s D and F tests also provided evidence for balancing selection in global PfAMA-1. These results collectively suggest that domains I and III of Myanmar PfAMA-1 are highly polymorphic with strong selection force acting on these domains, similar to other global PfAMA-1. Meiotic recombination between the adjacent polymorphic sites is one of main forces that drive allelic diversity in *Plasmodium* parasites [40]. Substantial levels of recombination events in PfAMA-1 from different geographical isolates have been reported previously [40, 43, 45–49]. Recombination events within Myanmar PfAMA-1 and decline of LD index R^2^ with the increase of nucleotide distance of the sequences evidenced meiotic recombination in Myanmar PfAMA-1, consistent with previous studies [40, 43, 45–49]. Higher values of recombination events were found in African PfAMA-1 sequences (Ghana and Tanzania) than those of PfAMA-1 from other geographical areas, suggesting that African PfAMA-1 might have more opportunity to have recombination events than other geographical PfAMA-1 probably due to high multiclonal infections, subsequent cross fertilization, and recombination in mosquitoes. High values of recombination parameters and very rapid decay of LD detected in global PfAMA-1 are independent and concordant indicators of high meiotic recombination rate in global PfAMA-1, supporting the notion that recombination is another important factor generating genetic diversity in global PfAMA-1.

Fst is a measure of population substructure and is one of the most useful tools for analysing the overall genetic differentiation among populations. Fst values at each locus are considered as no differentiation (0), low genetic differentiation (0–0.05), moderate differentiation (0.05–0.15), or high differentiation (0.15–0.25) [50]. Fst values for Myanmar PfAMA-1 in comparison with PfAMA-1 from other different geographical populations showed moderate levels of genetic differentiation, ranging from 0.058281 to 0.09740. Fst values between PfAMA-1 populations belonging to the same geographical area were relatively low. In particular, the value between Ghana and Tanzania was very low (0.00019), suggesting very low genetic differentiation among these two populations. However, Vanuatu showed relatively high Fst values for Ghana (0.12425) and Tanzania (0.14141), providing evidence for geographical isolation followed by population subdivision in these regions. Overall Fst values between global PfAMA-1 were in moderate differentiation ranges, indicating limited genetic differentiation of PfAMA-1 among global parasite populations.

The significant ratio of dN/dS and evidence of positive natural selection identified in PfAMA-1 of Myanmar and the other seven *P. falciparum* populations suggest that amino acid replacements are generally favored in PfAMA-1 and this adaptive evolution is presumably due to host immune pressure. Previous studies have suggested that polymorphic amino acid residues identified in PfAMA-1 are located on one side of the molecule, thereby exposing them to the exterior environment more easily with more access to host immune responses [40, 43, 51]. A recent study has provided information on potential RBC-binding regions, B-cell epitopes, and IURs across the ectodomain of PfAMA-1 [40]. To assess the association between natural selection and host immune pressure for PfAMA-1, genetic polymorphisms in predicted RBC-binding sites, B-cell epitopes, and IUR regions were analysed. Most amino acid changes found in global PfAMA-1 were predicted to be localized at the predicted RBC-binding sites, B-cell epitopes, or IUR regions of PfAMA-1. Eight of 11 predicted B-cell epitopes were polymorphic. In particular, B-cell epitopes 3, 4, 5, 8, and 10 (corresponding to domain I, junction between domain II and III, and domain III) showed high levels of nucleotide diversity in global PfAMA-1 analysed. Tajima’s D values for these predicted B-cells epitopes also suggested that these epitopes were under natural selection. The C1-L cluster, which located near the hydrophobic pocket of domain I in PfAMA-1, affects binding capacity of inhibitory monoclonal antibody, thereby contributing to antibody escape [52–55]. Global PfAMA-1 analysed in this study had clustering amino acid polymorphisms, including tri- and hepta-morphic changes in C1-L region. This supports the notion that strong natural selection force which generates genetic diversity of PfAMA-1, thereby contributing to host immune escape acts on this region [21, 54, 55]. Meanwhile, only a few less frequent dimorphic amino acid changes were identified in loop II of global PfAMA-1, the target of 4G2 inhibitory antibody [56], coinciding with results of a previous study [40]. High degree of amino acid sequence conservation of the loop II among global PfAMA-1 further supports the hypothesis that amino acid sequences in this region are subjected to strong natural selection. Therefore, this region might be useful as a component of PfAMA-1-based vaccine.

To develop a globally effective malaria vaccine based on PfAMA-1, it would be important to understand whether PfAMA-1 haplotypes with distinct clusters are antigenically different. Haplotype network analysis of 517 global PfAMA-1 sequences suggested that a total of 174 distinct haplotypes were identified among global population. No haplotype that fully covers haplotypes from all geographical areas analyzed in this study was identified. PfAMA-1 based vaccines currently studied are based on sequences of most extensively studied forms of *P. falciparum*, 3D7 and FVO [57–59]. However, only 13.7% of global PfAMA-1 haplotypes analysed in this study was identical to the FVO. The frequency of global PfAMA-1 haplotypes with identical sequence to 3D7 was much lower. These raised a concern that vaccine based on these two forms of haplotypes may not offer effective protection for global *P. falciparum* isolates. A recent report suggested that PfAMA-1 sequence variations may not necessarily be strong predictors of antigenic differences or the level of cross-inhibitory antibody activity because not all polymorphic residues contribute equally to antibody binding and escape [60]. Antigenic diversity of PfAMA-1 is limited and a vaccine targeting a small number of PfAMA-1 alleles might be sufficient for coverage against naturally-circulating *P. falciparum* populations in different endemic areas [27]. The results of this study also suggested that the genetic diversity is relatively limited among global PfAMA-1, even though substantial geographic differentiations were also identified among the populations. However, global PfAMA-1 is under natural selection and high level of meiotic recombination that can produce new alleles occurs in the gene population. And therefore, a multi-allele approach for developing PfAMA-1 based malaria vaccines should be considered to maximize vaccine efficacy.

## Conclusions

Overall patterns of nucleotide diversity and distributions of amino acid changes found in Myanmar PfAMA-1 were similar to those from global PfAMA-1, albeit several amino acid changes were identified only in Myanmar PfAMA-1. Balancing natural selection across PfAMA-1 and high degree of recombination events observed in Myanmar and global PfAMA-1 suggest that natural selection and intragenic recombination might be main driving forces that generate genetic diversity in global PfAMA-1. Most common amino acid changes found in global PfAMA-1 were located at predicted B-cell epitopes. High degree of nucleotide diversity and balancing natural selection of the B-cell epitope regions suggest that strong natural selection might have act on epitopes associated with host immune pressure for PfAMA-1. Limited genetic differentiation of PfAMA-1 was estimated between and among global parasite populations, even though highly complicated haplotype diversity was identified in global population. However, global PfAMA-1 is under natural selection and high level of meiotic recombination that can produce new alleles occurs in the gene population.

The results of this study warrant continuous examination of PfAMA-1 nucleotide and amino acid variations in global PfAMA-1 to better understand the polymorphic nature of PfAMA-1 and design effective vaccine against global *P. falciparum* populations.

## References

[1] WHO (2016). World malaria report 2016.

[2] Richards JS, Beeson JG (2009). The future for blood-stage vaccines against malaria. Immunol Cell Biol.

[3] Florens L, Washburn MP, Raine JD, Anthony RM, Grainger M, Haynes JD (2002). A proteomic view of the *Plasmodium falciparum* life cycle. Nature.

[4] Takala SL, Plowe CV (2009). Genetic diversity and malaria vaccine design, testing and efficacy: preventing and overcoming ‘vaccine resistant malaria. Parasite Immunol.

[5] Ferreira MU, da Silva Nunes M, Wunderlich G (2004). Antigenic diversity and immune evasion by malaria parasites. Clin Diagn Lab Immunol.

[6] Escalante AA, Lal AA, Ayala FJ (1998). Genetic polymorphism and natural selection in the malaria parasite *Plasmodium falciparum*. Genetics.

[7] Healer J, Crawford S, Ralph S, McFadden G, Cowman AF (2002). Independent translocation of two micronemal proteins in developing *Plasmodium falciparum* merozoites. Infect Immun.

[8] Silvie O, Franetich JF, Charrin S, Mueller MS, Siau A, Bodescot M (2004). A role for apical membrane antigen 1 during invasion of hepatocytes by *Plasmodium falciparum* sporozoites. J Biol Chem.

[9] Peterson MG, Marshall VM, Smythe JA, Crewther PE, Lew A, Silva A, Anders RF, Kemp DJ (1989). Integral membrane protein located in the apical complex of *Plasmodium falciparum*. Mol Cell Biol.

[10] Mitchell GH, Thomas AW, Margos G, Dluzewski AR, Bannister LH (2004). Apical membrane antigen 1, a major malaria vaccine candidate, mediates the close attachment of invasive merozoites to host red blood cells. Infect Immun.

[11] Yap A, Azevedo MF, Gilson PR, Weiss GE, O’Neill MT, Wilson DW (2014). Conditional expression of apical membrane antigen 1 in *Plasmodium falciparum* shows it is required for erythrocyte invasion by merozoites. Cell Microbiol.

[12] Hodder AN, Crewther PE, Matthew ML, Reid GE, Moritz RL, Simpson RJ (1996). The disulfide bond structure of Plasmodium apical membrane antigen-1. J Biol Chem.

[13] Udhayakumar V, Kariuki S, Kolczack M, Girma M, Roberts JM, Oloo AJ (2001). Longitudinal study of natural immune responses to the *Plasmodium falciparum* apical membrane antigen (AMA-1) in a holoendemic region of malaria in western Kenya: Asembo Bay Cohort Project VIII. Am J Trop Med Hyg.

[14] Cortés A, Mellombo M, Masciantonio R, Murphy VJ, Reeder JC, Anders RF (2005). Allele specificity of naturally acquired antibody responses against *Plasmodium falciparum* apical membrane antigen 1. Infect Immun.

[15] Rodrigues MH, Rodriques KM, Oliveira TR, Cômodo AN, Rodriques MM, Kocken CH (2005). Antibody response of naturally infected individuals to recombinant *Plasmodium vivax* apical membrane antigen-1. Int J Parasitol.

[16] Moncunill G, Mayor A, Jiménez A, Nhabomba A, Casas-Vila N, Puyol L (2013). High antibody responses against *Plasmodium falciparum* in immigrants after extended periods of interrupted exposure to malaria. PLoS ONE.

[17] Gentil F, Bargieri DY, Leite JA, Françoso KS, Patricio MB, Espíndola NM (2010). A recombinant vaccine based on domain II of *Plasmodium vivax* apical membrane antigen 1 induces high antibody titres in mice. Vaccine.

[18] Remarque EJ, Faber BW, Kocken CH, Thomas AW (2008). Apical membrane antigen 1: a malaria vaccine candidate in review. Trends Parasitol.

[19] Thera MA, Coulibaly D, Kone AK, Guindo AB, Traore K, Sall AH (2016). Phase 1 randomized controlled trial to evaluate the safety and immunogenicity of recombinant Pichia pastoris-expressed *Plasmodium falciparum* apical membrane antigen 1 (PfAMA1-FVO [25-545]) in healthy Malian adults in Bandiagara. Malar J.

[20] Osier FH, Fegan G, Polley SD, Murungi L, Verra F, Tetteh KK, Lowe B (2008). Breadth and magnitude of antibody responses to multiple *Plasmodium falciparum* merozoite antigens are associated with protection from clinical malaria. Infect Immun.

[21] Mugyenyi CK, Elliott SR, McCallum FJ, Anders RF, Marsh K, Beeson JG (2013). Antibodies to polymorphic invasion-inhibitory and non-inhibitory epitopes of *Plasmodium falciparum* apical membrane antigen 1 in human malaria. PLoS ONE.

[22] Polley SD, Mwangi T, Kocken CH, Thomas AW, Dutta S, Lanar DE (2004). Human antibodies to recombinant protein constructs of *Plasmodium falciparum* apical membrane antigen1 (AMA1) and their associations with protection from malaria. Vaccine.

[23] Escalante AA, Grebert HM, Chaiyaroj SC, Magris M, Biswas S, Nahlen BL (2001). Polymorphism in the gene encoding the apical membrane antigen-1 (AMA-1) of *Plasmodium falciparum*. X. Asembo Bay Cohort Project. Mol Biochem Parasitol.

[24] Polley SD, Conway DJ (2001). Strong diversifying selection on domains of the *Plasmodium falciparum* apical membrane antigen 1 gene. Genetics.

[25] Moon SU, Na BK, Kang JM, Kim JY, Cho SH, Park YK (2010). Genetic polymorphism and effect of natural selection at domain I of apical membrane antigen-1 (AMA-1) in *Plasmodium vivax* isolates from Myanmar. Acta Trop.

[26] Arnott A, Mueller I, Ramsland PA, Siba PM, Reeder JC, Barry AE (2013). Global population structure of the genes encoding the malaria vaccine candidate, *Plasmodium vivax* apical membrane antigen 1(PvAMA1). PLoS Negl Trop Dis.

[27] Terheggen U, Drew DR, Hodder AN, Cross NJ, Mugyenyi CK, Barry AE (2014). Limited antigenic diversity of *Plasmodium falciparum* apical membrane antigen 1 supports the development of effective multi-allele vaccines. BMC Med.

[28] Kang JM, Lee J, Cho PY, Moon SU, Ju HL, Ahn SK (2015). Population genetic structure and natural selection of apical membrane antigen-1 in *Plasmodium vivax* Korean isolates. Malar J.

[29] Volkman SK, Ndiaye D, Diakite M, Koita OA, Nwakanma D, Daniels RF (2012). Application of genomics to field investigations of malaria by the international centers of excellence for malaria research. Acta Trop.

[30] WHO (2010). Malaria in the Greater Mekong Subregion: regional and country profiles.

[31] Kang JM, Cho PY, Moe M, Lee J, Jun H, Lee HW (2017). Comparison of the diagnostic performance of microscopic examination with nested polymerase chain reaction for optimum malaria diagnosis in Upper Myanmar. Malar J.

[32] Tun KM, Imwong M, Lwin KM, Win AA, Hlaing TM, Hlaing T (2015). Spread of artemisinin-resistant *Plasmodium falciparum* in Myanmar: a cross-sectional survey of the K13 molecular marker. Lancet Infect Dis.

[33] Snounou G, Singh B (2002). Nested PCR analysis of *Plasmodium* parasites. Methods Mol Med.

[34] Librado P, Rozas J (2009). DnaSP v5: a software for comprehensive analysis of DNA polymorphism data. Bioinformatics.

[35] Tamura K, Dudley J, Nei M, Kumar S (2007). MEGA4: molecular evolutionary genetics analysis (MEGA) software version 4.0. Mol Biol Evol.

[36] Nei M, Gojobori T (1986). Simple methods for estimating the numbers of synonymous and nonsynonymous nucleotide substitutions. Mol Biol Evol.

[37] Tajima F (1989). Statistical method for testing the neutral mutation hypothesis by DNA polymorphism. Genetics.

[38] Fu YX, Li WH (1993). Statistical tests of neutrality of mutations. Genetics.

[39] Bandelt HJ, Forster P, Röhl A (1999). Median-joining networks for inferring intraspecific phylogenies. Mol Biol Evol.

[40] Mehrizi AA, Sepehri M, Karimi F, Djadid ND, Zakeri S (2013). Population genetics, sequence diversity and selection in the gene encoding the *Plasmodium falciparum* apical membrane antigen 1 in clinical isolates from the south-east of Iran. Infect Genet Evol.

[41] Urquiza M, Suarez JE, Cardenas C, Lopez R, Puentes A, Chavez F (2000). *Plasmodium falciparum* AMA-1 erythrocyte binding peptides implicate AMA-1 as erythrocyte binding protein. Vaccine.

[42] Cortés A, Mellombo M, Mueller I, Benet A, Reeder JC, Anders RF (2003). Geographical structure of diversity and differences between symptomatic and asymptomatic infections for *Plasmodium falciparum* vaccine candidate AMA1. Infect Immun.

[43] Basu M, Maji AK, Mitra M, Sengupta S (2013). Natural selection and population genetic structure of domain-I of *Plasmodium falciparum* apical membrane antigen-1 in India. Infect Genet Evol.

[44] Al-Qahtani AA, Abdel-Muhsin AM, Bin Dajem SM, AlSheikh AA, Bohol MF, Al-Ahdal MN (2016). Comparative sequence analysis of domain I of *Plasmodium falciparum* apical membrane antigen 1 from Saudi Arabia and worldwide isolates. Infect Genet Evol.

[45] Zhu X, Zhao Z, Feng Y, Li P, Liu F, Liu J (2016). Genetic diversity of the *Plasmodium falciparum* apical membrane antigen I gene in parasite population from the China–Myanmar border area. Infect Genet Evol.

[46] Eisen DP, Marshall VM, Billman-Jacobe H, Coppel RL (1999). A *Plasmodium falciparum* apical membrane antigen-1 (AMA-1) gene apparently generated by intragenic recombination. Mol Biochem Parasitol.

[47] Garg S, Alam MT, Das MK, Dev V, Kumar A, Dash AP (2007). Sequence diversity and natural selection at domain I of the apical membrane antigen 1 among Indian *Plasmodium falciparum* populations. Malar J.

[48] Quang ND, Hoa PT, Tuan MS, Viet NX, Jalloh A, Matsuoka H (2009). Polymorphism at the apical membrane antigen 1 gene (AMA1) of the malaria parasite *Plasmodium falciparum* in a Vietnamese population. Biochem Genet.

[49] Mardani A, Keshavarz H, Heidari A, Hajjaran H, Raeisi A, Khorramizadeh MR (2012). Genetic diversity and natural selection at the domain I of apical membrane antigen-1 (AMA-1) of *Plasmodium falciparum* in isolates from Iran. Exp Parasitol.

[50] Balloux F, Lugon-Moulin N (2002). The estimation of population differentiation with microsatellite markers. Mol Ecol.

[51] Bai T, Becker M, Gupta A, Strike P, Murphy VJ, Anders RF, Batchelor AH (2005). Structure of AMA1 from *Plasmodium falciparum* reveals a clustering of polymorphisms that surround a conserved hydrophobic pocket. Proc Natl Acad Sci USA.

[52] Coley AM, Parisi K, Masciantonio R, Hoeck J, Casey JL, Murphy VJ (2006). The most polymorphic residue on *Plasmodium falciparum* apical membrane antigen 1 determines binding of an invasion-inhibitory antibody. Infect Immun.

[53] Coley AM, Gupta A, Murphy VJ, Bai T, Kim H, Foley M (2007). Structure of the malaria antigen AMA1 in complex with a growth inhibitory antibody. PLoS Pathog.

[54] Dutta S, Lee SY, Batchelor AH, Lanar DE (2007). Structural basis of antigenic escape of a malaria vaccine candidate. Proc Natl Acad Sci USA.

[55] Harris KS, Adda CG, Khore M, Drew DR, Valentini-Gatt A, Fowkes FJ (2014). Use of immunodampening to overcome diversity in the malarial vaccine candidate apical membrane antigen 1. Infect Immun.

[56] Collins CR, Withers-Martinez C, Bentley GA, Batchelor AH, Thomas AW, Blackman MJ (2007). Fine mapping of an epitope recognized by an invasion inhibitory monoclonal antibody on the malaria vaccine candidate apical membrane antigen 1. J Biol Chem.

[57] Thera MA, Doumbo OK, Coulibaly D, Laurens MB, Kone AK, Guindo AB (2010). Safety and immunogenicity of an AMA1 malaria vaccine in Malian children: results of a phase 1 randomized controlled trial. PLoS ONE.

[58] Thera MA, Doumbo OK, Coulibaly D, Laurens MB, Ouattara A, Kone AK (2011). A field trial to assess a blood-stage malaria vaccine. N Engl J Med.

[59] Laurens MB, Thera MA, Coulibaly D, Ouattara A, Kone AK, Guindo AB (2013). Extended safety, immunogenicity and efficacy of a blood-stage malaria vaccine in malian children: 24-month follow-up of a randomized, double blinded phase 2 trial. PLoS ONE.

[60] Drew DR, Hodder AN, Wilson DW, Foley M, Mueller I, Siba PM (2012). Defining the antigenic diversity of *Plasmodium falciparum* apical membrane antigen 1 and the requirements for a multi-allele vaccine against malaria. PLoS ONE.

